# Volatile Constituents and Antioxidant Activity of Peel, Flowers and Leaf Oils of *Citrus aurantium* L. Growing in Greece

**DOI:** 10.3390/molecules180910639

**Published:** 2013-09-02

**Authors:** Eirini Sarrou, Paschalina Chatzopoulou, Kortessa Dimassi-Theriou, Ioannis Therios

**Affiliations:** 1 Laboratory of Pomology, School of Horticulture, Aristotle University of Thessaloniki 54124, Greece; E-Mails: dimassik@agro.auth.gr (K.D.-T.); therios@agro.auth.gr (I.T.); 2 Hellenic Agricultural Organization - Demeter (former NAGREF), Department of Aromatic and Medicinal Plants, Thessaloniki 57001, Greece; E-Mail: chatzopoulou@nagref.gr

**Keywords:** antioxidant activity, *Citrus aurantium* L., essential oils composition, neroli, peel, petitgrain

## Abstract

The volatile constituents of the essential oils of the peel, flower (neroli) and leaves (petitgrain) of bitter orange (*Citrus aurantium* L.) growing in Greece were studied by GC-MS. The analytical procedures enabled the quantitative determination of 31 components. More specifically, the components of the essential oils identified were: twelve in the peel, twenty-six in the flowers, and twenty and sixteen in old and young leaves, respectively. The major constituents of the different parts of *Citrus aurantium* L. essential oils were: β-pinene (0.62%–19.08%), limonene (0.53%–94.67%), *trans*-β-ocimene (3.11%–6.06%), linalool (0.76%–58.21%), and α-terpineol (0.13%–12.89%). The DPPH test demonstrated that the essential oils in the old leaves had the maximum antioxidant activity, followed by the flowers, young leaves and the peel in that order. This study updates the data in the literature on the essential oils of bitter orange, and provides information on the composition of the oils for a further evaluation of this product.

## 1. Introduction

*Citrus aurantium* L. (Rutaceae family), also known as sour orange or bitter orange, is generally used as a rootstock and has a number of advantages, including resistance to several viral diseases, tolerance to cold, improvement in the fruit quality of the grafted plants, and it can be used as an ornamental tree [1]. In addition, *Citrus aurantium* L. is among the species that have been used for medicinal purposes on account of the various bioactive compounds that it contains, such as phenolics, flavonoids, essential oils, and vitamins. The parts mostly used for medicinal purposes are the fruit peel, the flowers and the leaves of the plant [2]. Immature fruits are sometimes pickled or used as a condiment. The peel of *C. aurantium* L. contains limonene as the major essential oil constituent, flavonoids, hesperidin, neohesperidin, naringin and tangaretin and it is often used in marmalade production, whereas dried peel is used in bouquet garni and for flavoring the Belgian beer called Orange Muscat [3]. Essential oils from the dried peel of unriped *Citrus aurantium* L. fruits flavor drinks and liquors, like Curaçao, Cointreau, and Triple Sec. The flowers are used in teas, whereas the essential oil (neroli) is used in perfumes, liqueurs, and orange-flower water used to flavor sweets [4]. Besides the uses mentioned above, undiluted essential oils are sold at a high price on the international market of aromatherapy, perfume and cosmetic industry as well. Moreover, numerous studies have been reported on the assorted medical uses (antioxidant, antimicrobial, antifungal, antiparasitic, anti-inflammatory, etc.) of several *C. aurantium* essential oil constituents and other compounds isolated from peel [5,6,7]. Limonene and β-myrcene were reported as the major components of the peel oil while linalool, linalyl acetate and α-terpineol predominate in the leaf oil (petitgrain) [8].

The aim of this study was the characterization of the essential oils from different parts (flowers, peel and leaves) of the *Citrus aurantium* L. trees growing in Northern Greece and to investigate their antioxidant activity. To our knowledge this is the first report on the essential oils obtained from different parts of Greek *Citrus aurantium* L. 

## 2. Results and Discussion

### 2.1. Essential Oil Composition

A clear yellow volatile oil with a fresh sweet odor was obtained through the hydrodistillation of flowers, peel, young and old leaves of *C. aurantium* L. at 0.12%, 1.67%, 0.27% and 0.45% respectively (mL/100 g of fresh tissue). The chemical composition of the essential oils from different parts of *C. aurantium* L. was analyzed by GC-MS. Qualitative and quantitative analytical results are shown in Table 1.

In neroli oil twenty six constituents accounted for the 99.44% of the oil. The major compounds were linalool (29.14%), β-pinene (19.08%), limonene (12.04%), *trans***-**β**-**ocimene (6.06%) and *E*-farnesol (5.14%). Twenty components constituted 99.87% of the peel oil (Table 1). The main compounds were: limonene (94.67%), myrcene (2.00%), linalool (0.76%), β-pinene (0.62%) and α-pinene (0.53%).

**Table 1:** Essential oil content (%) of flowers, peel, young and old leaf of *Citrus aurantium* L. and their main constituents.

Nr	Compounds ^a^	*RT* ^b^	*Retention Index* ^c^	Concentration % ^d,e^			
				Flowers	Peel	Young Leaves	Old Leaves
1	α-Pinene	7.722	934 (939)	1.35 ± 0.01	0.53 ± 0.02	0.19 ± 0.00	-
2	Sabinene	9.542	973 (976)	2.01 ± 0.20	0.18 ± 0.01	0.37 ± 0.00	0.22 ± 0.03
3	β-Pinene	9.750	980 (980)	19.08 ± 0.18	0.62 ± 0.04	3.58 ± 0.01	1.90 ± 0.31
4	Myrcene	10.509	991 (991)	1.59 ± 0.01	2.00 ± 0.04	1.63 ± 0.06	2.74 ± 0.32
5	Octanal	10.953	999 (1001)	-	0.24 ± 0.01	-	-
6	3-δ-Carene	11.816	1014 (1011)	0.17 ± 0.00	-	-	-
7	Limonene	12.630	1029 (1031)	12.04 ± 0.16	94.67 ± 0.01	0.53 ± 0.01	0.77 ± 0.09
8	*cis*-β-Ocimene	13.206	1039 (1040)	0.77 ± 0.02	0.30 ± 0.01	0.71 ± 0.02	1.22 ± 0.07
9	*trans*-β-Ocimene	13.884	1049 (1050)	6.06 ± 0.01	-	4.08 ± 0.05	3.11 ± 0.21
10	γ-Terpinene	14.550	1060 (1062)	0.36 ± 0.01	-	-	-
11	Linalool oxide	15.353	1071 (1074)	0.29 ± 0.02	-	-	-
12	α-Terpinolene	16.440	1086 (1088)	0.47 ± 0.01	-	0.40 ± 0.01	0.70 ± 0.04
13	Linalool	17.729	1102 (1104)	29.14 ± 0.38	0.76 ± 0.04	58.21 ± 0.37	36.03 ± 0.60
14	Terpin 4-ol	23.122	1174 (1177)	0.68 ± 0.02	-	0.17 ± 0.01	0.13 ± 0.02
15	α-Terpineol	24.267	1187 (1189)	4.56 ± 0.05	0.13 ± 0.01	7.11 ± 0.06	12.89 ± 0.37
16	Decanal	25.574	1202 (1204)	-	0.16 ± 0.03	-	-
17	Nerol	27.489	1227 (1228)	0.83 ± 0.02	-	1.45 ± 0.02	2.89 ± 0.06
18	Geraniol	29.864	1257 (1255)	4.31 ± 1.43	-	-	-
19	Linalyl acetate	30.963	1259 (1257)	3.88 ± 0.40	0.18 ± 0.03	12.42 ± 0.13	23.00 ± 1.42
20	Methyl anthranylate	36.245	1334 (1337)	0.19 ± 0.00	-	-	-
21	δ-Elemene	36.458	1337 (1339)	0.12 ± 0.00	-	-	-
22	Terpinyl acetate	37.426	1349 (1350)	0.20 ± 0.00	-	-	0.11 ± 0.00
23	Neryl acetate	38.901	1368 (1365)	1.30 ± 0.01	0.10 ± 0.00	2.18 ± 0.07	4.46 ± 0.22
24	Geranyl acetate	40.419	1386 (1383)	2.59 ± 0.04	-	4.49 ± 0.11	8.70 ± 0.37
25	β-Caryophyllene	42.266	1412 (1418)	0.42 ± 0.01	-	1.09 ± 0.02	0.22 ± 0.01
26	α-Humulene	44.268	1447 (1454)	-	-	0.10 ± 0.00	-
27	(*E*)-β-Farnesene	44913	1458 (1458)	-	-	0.13 ± 0.01	-
28	δ-Germacrene	45.948	1476 (1480)	0.13 ±0.01	-	-	-
29	Bicyclogermacrene	46.764	1489 (1494)	-	-	0.18 ± 0.00	0.20 ± 0.01
30	(*E*)-Nerolidol	50.451	1566 (1564)	1.76 ± 0.03	-	-	0.10 ± 0.00
31	(*E*)-Farnesol	55.544	1725 (1722)	5.14 ± 0.02	-	-	-
	Total percentage ^d^			99.44%	99.87%	99.02%	99.39%
	Essential oil (%) content			0.12 ± 0.01	1.67 ± 0.07	0.27 ± 0.01	0.45 ± 0.02

^a,b^ order of elution on DB-5 column, RT: retention time; ^c^ Calculated relative to C7-C40 n-alcanes, on DB-5 column. In parentheses, Literature Retention Indices on similar column; ^d^ Percentage of the total peak area. Components with percentage ≥ 0.1% are presented; ^e^: ± St error.

The qualitative composition of both petitgrain oils (obtained from young and old leaves) was similar. Sixteen compounds were common, among which the major components were: linalool (58.21%–36.03%), α-terpineol (7.11%–12.89%), geranyl acetate (4.49%–8.70%), neryl acetate (2.18%–4.46%) and *trans*-β-ocimene (4.08%–3.11%). α-Pinene, α-humulene and (*E*)-β-farnesene (amounting to 0.42%) were detected only in the young leaf oil, whereas terpinyl acetate and nerolidol E (cumulative 0.21%) were detected in the old leaf oil.

**Figure 1:**
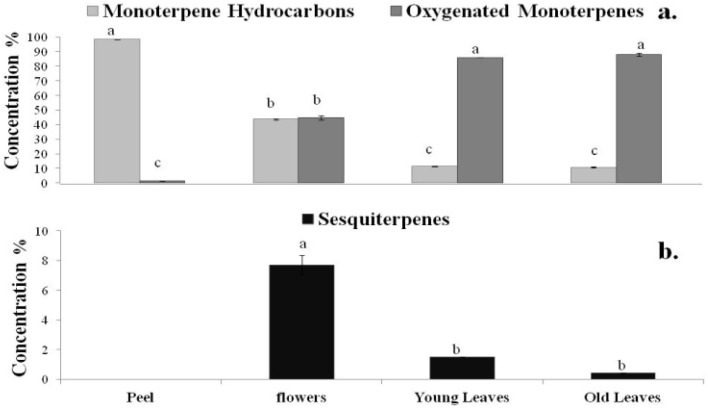
(**a**) % yield of monoterpene hydrocarbons, oxygenated monoterpenes and (**b**) sesquiterpenes, in the oils from different parts of *Citrus aurantium* L. Vertical bars represent means (n = 3) ± SE. Different letters indicate a significant difference at *p* ≤ 0.05.

There were significant differences in the qualitative and quantitative composition of the examined oils. Peel oil is composed almost of monoterpene hydrocarbons (98.30%), mainly limonene, while oxygenated monoterpenes are dominant compounds of leaf and flowers oils, detected at higher amounts in old leaves (88.09%) (Figure 1a). The amount of sesquiterpenes in neroli oil (7.67%) differed significantly from petitgrain oils, while in the peel oil they were not detected at all (Figure 1b).

Several studies on the chemical composition of the essential oils extracted from *C. aurantium* L. seem to be in agreement with our findings, indicating that the main components of peel oil are monoterpene hydrocarbons like limonene and myrcene, while those of petitgrain oil are linalool, linalyl acetate, geranyl acetate and α-terpineol [8,9,10,11,12,13]; and neroli oil contained linalool, limonene and linalyl acetate [14,15,16]. Limonene, estimated at 96.7%, was also the main component of *C. aurantium* peel oil from Greece [17]. Quantitative differences may arise due to different genotype, the pedoclimatic conditions in the growing areas, etc.

### 2.2. DPPH Radical Scavenging Activity

**Figure 2:**
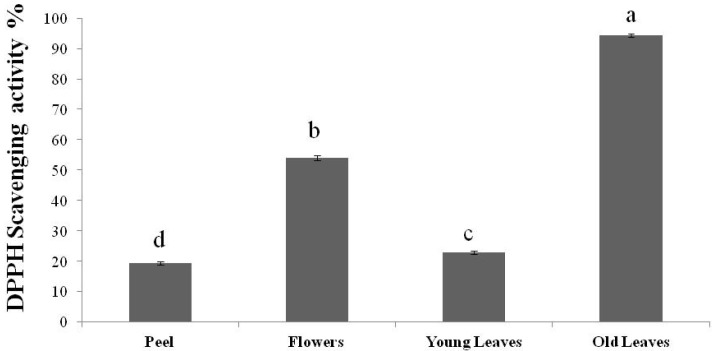
DPPH scavenging activity of the essential oils obtained from peel, flowers, young and old leaves. Vertical bars represent means (n = 3) ± SE. Different letters indicate a significant difference at *p* ≤ 0.05.

The ability of the essential oils to act as hydrogen or electron donors in the transformation of DPPH into its reduced form DPPH-H was investigated. The antioxidant activities of all the oils tested are presented in Figure 2. All the essential oils were able to reduce the stable, purple-colored radical DPPH to the yellow-colored DPPH-H. The scavenging activity of flowers, young leaves and peel oil was determined at 53.98%, 22.79% and 19.29%, respectively. The oil of the old leaves showed the highest scavenging activity (94.36%). This could be due to water losses of the older leaves which affects the accumulation of the secondary metabolites through the reallocation of the assimilated carbon as leaf growth is progressively reduced. Another possible explanation is that some components of the above oils contribute differently to the antioxidant activity.

Several studies on the chemical composition and bioactivity of different citrus oils reported strong radical scavenging activity [18,19,20,21]. It is suggested that, even at low concentrations, authentic flavor components such as γ-terpinene, terpinolene, geraniol, β-pinene and myrcene have high antioxidant activities [7,22]. Choi *et al*. found that the radical scavenging activity of 34 kinds of citrus essential oils on DPPH ranged from 17.7% to 64%. These activities were found to be higher when the oils contained geraniol, terpinolene and γ-terpinene [7]. The fact that the essential oil of old leaves and flowers had the highest scavenging activity, may be due to the presence of α-terpinolene and nerol in petitgrain oil and γ-terpinene, nerol and geraniol in the neroli oil [7,22]. Nevertheless, further studies are needed to evaluate the scavenging effects of the authentic compounds of *C. aurantium* essential oils. However, the bioactivity of the essential oils generally results from a complex interaction between its constituents, which produce both synergistic and antagonistic responses [23].

## 3. Experimental

### 3.1. Chemicals

The *n***-**alkanes (C_7_-C_40_) was purchased from Supelco (Bellefonte, PA, USA). Anhydrous sodium sulphate (Na_2_SO_4_) and 2.2-diphenyl-1-picrylhydrazyl (DPPH) used were purchased from Roth and Sigma-Aldrich (Steinheim, Germany). The highest purity pentane used for the GC-MS was purchased from Panreac Quimica S.L.U (Barcelona, Spain).

### 3.2. Plant Material

Different parts of mature *Citrus aurantium* L. trees were collected from the same orchard, in Thessaloniki, Greece, (40°34'35'' N 22°57'19'' E), 2011. At the beginning of April, young leaves (up to the 3rd node from the top of the shoots) and old leaves (from the basal parts of the shoots) were gathered between 11:00–12:00 in the morning and they were then cut in half. At the same time the flowers were also picked. The fresh fruits, on the other hand, were collected at the beginning of October. As soon as each part was gathered, it was immediately subjected to hydrodistillation.

### 3.3. Determination of Essential Oils

The essential oil content was determined using the European Pharmacopoeia apparatus (Clevenger-type). The various parts of *Citrus aurantium* L. were subjected to hydrodistillation as follows: the fresh leaves for 4 h, the flowers for 3 h, and the peel also for 3 h, with a distillation rate 3 to 3.5 mL/min. Hydrodistilled fresh mass was 60 g for the leaves, 150 g and 35 g for the flowers and the peel respectively. The oil yields were estimated on the basis of the wet weight plant material (mL/100 g). The obtained essential oils were then dried over anhydrous sodium sulphate and stored at 4–6 °C.

### 3.4. GC-MS Analysis

The GC-MS analysis was performed on a DB-5 column, using a Gas Chromatograph Shimadzu GC-17A interfaced with a Mass Spectrometer Shimadzu QP-5050A supported by the Class 5000 software. Injection temperature: 260 °C, interface heating: 300 °C, ion source heating: 200 °C, EI mode: 70 eV, scan range: 41–450 amu, and scan time 0.50 s. Oven temperature programs: (a) 55–120 °C (3 °C/min), 120–200 °C (4 °C/min), 200–220 °C (6 °C/min) and 220 °C for 5 min and (b) 60–240 °C at 3 °C/min, carrier gas He, 54.8 kPa, split ratio 1:30. The relative content of each compound was calculated as percent of the total chromatographic area and the results are expressed as means of triplicate experiments.

### 3.5. Identification of Components

The identification of the components was based on comparison of their retention indices (RI) relative to *n*-alkanes (C_7_-C_40_), with corresponding literature data [24,25,26] as well by matching (a) their spectra with those from MS libraries (NIST 98, Willey, Adams 1995) [26] and (b) the retention time (RT) of co-eluting reference compounds-peak enrichment technique (authentic samples obtained from Roth and Sigma Aldrich).

### 3.6. DPPH Radical Scavenging Activity Assay

Total antioxidant activity was determined following the method of Su *et al*. [27]. Fifty µL of essential oils (100% concentration) added to 0.1 mM DPPH solution (2.95 mL). After 1 h the absorbance of the reaction mixture was measured in triplicate at 517 nm on a spectrophotometer (Prim, SECOMAM, Domont, France). The control solution was prepared by adding absolute ethanol (50 µL) to the DPPH solution and ethanol was used as a blank. Measurements were expressed as scavenging activity %. The antioxidant activity was determined by the following formula:

Scavenging Activity (%) = {(Abs *control* − Abs *sample*)/Abs *control*} *×* 100 (1)

where Abs is the absorbance at 517 nm.

### 3.7. Statistical Analysis

All samples were analyzed in triplicate and the results are expressed as the means. The data were analysed with Analysis of Variance (ANOVA), using the statistical package SPSS 17.0 (SPSS Inc., Chicago, IL, USA). For mean comparison, the Duncan’s multiple range test and standard error (S.E) were used at *p* ≤ 0.05 to establish significant differences.

## 4. Conclusions

In conclusion, our study indicated that Greek *Citrus aurantium* L. essential oils are a potential natural source of monoterpenes such as limonene, β-pinene, linalool and linalyl acetate. In addition, as a result of the scavenging effects, it is expected that citrus essential oils and the related flavor components may contribute to the prevention of oxidation as antioxidants and free radical scavengers. This research may be of interest from a functional point of view and for the valorization of *Citrus aurantium* L. in Greece and the wider Mediterranean region.
